# A genetic linkage map of *Venturia inaequalis*, the causal agent of apple scab

**DOI:** 10.1186/1756-0500-2-163

**Published:** 2009-08-18

**Authors:** Xiangming Xu, Tony Roberts, Dez Barbara, Nick G Harvey, Liqiang Gao, Daniel J Sargent

**Affiliations:** 1 East Malling Research, New Road, East Malling, ME19 6BJ, UK; 2 Warwick HRI, University of Warwick, Wellesbourne, Warwick, CV35 9EF, UK; 3 College of Plant Protection, Northwest A&F University, Yangling, Shaanxi Province, PR China

## Abstract

**Background:**

*Venturia inaequalis *is an economically-important disease of apple causing annual epidemics of scab worldwide. The pathogen is a heterothallic ascomycete with an annual cycle of sexual reproduction on infected apple leaf litter, followed by several cycles of asexual reproduction during the apple growing season. Current disease control is achieved mainly through scheduled applications of fungicides. Genetic linkage maps are essential for studying genome structure and organisation, and are a valuable tool for identifying the location of genes controlling important traits of interest such as avirulence, host specificity and mating type in *V. inaequalis*. In this study, we performed a wide cross under *in vitro *conditions between an isolate of *V. inaequalis *from China and one from the UK to obtain a genetically diverse mapping population of ascospore progeny isolates and produced a map using AFLP and microsatellite (SSR) markers.

**Findings:**

Eighty-three progeny were obtained from the cross between isolates C0154 (China) × 01/213 (UK). The progeny was screened with 18 AFLP primer combinations and 31 SSRs, and scored for the mating type locus *MAT*. A linkage map was constructed consisting of 294 markers (283 AFLPs, ten SSRs and the *MAT *locus), spanning eleven linkage groups and with a total map length of 1106 cM. The length of individual linkage groups ranged from 30.4 cM (Vi-11) to 166 cM (Vi-1). The number of molecular markers per linkage group ranged from 7 on Vi-11 to 48 on Vi-3; the average distance between two loci within each group varied from 2.4 cM (Vi-4) to 7.5 cM (Vi-9). The maximum map length between two markers within a linkage group was 15.8 cM. The *MAT *locus was mapped to a small linkage group and was tightly linked to two AFLP markers. The map presented is over four times longer than the previously published map of *V. inaequalis *which had a total genetic distance of just 270 cM.

**Conclusion:**

A genetic linkage map is an important tool for investigating the genetics of important traits in *V. inaequalis *such as virulence factors, aggressiveness and mating type. The linkage map presented here represents a significant improvement over currently published maps for studying genome structure and organisation, and for mapping genes of economic importance on the *V. inaequalis *genome.

## Research hypothesis

*Venturia inaequalis *is a fungal pathogen of major economic importance, causing annual epidemics of apple scab [1]. The fungus is a haploid organism with seven chromosomes [2] and is a heterothallic ascomycete with an annual cycle of sexual reproduction on infected apple leaf litter, followed by several cycles of asexual reproduction during the apple growing season. High standards of fruit quality require very high levels of control, particularly in dessert cultivars, and these controls are currently achieved primarily through scheduled applications of fungicides.

For breeding apple cultivars with durable resistance to *V. inaequalis*, it is essential to understand pathogen virulence structures and the extent to which evolutionary forces may alter such structures. Analysis of microsatellite profiles of *V. inaequalis *samples from five continents suggested that the fungus originated in Central Asia, but it is now well established worldwide displaying high within-population diversity [3-5]. Isolates obtained from different apple cultivars planted in the same orchard in the UK differed significantly in their virulence characteristics [6] as well as at the molecular level, based on AFLP analysis, whereas isolates from different cultivars or regions in China did not [7]. More information on within-population variability is needed to understand better the forces acting on the host-pathogen co-evolution.

Genetic linkage maps are essential for studying genome structure and organisation, and are a valuable tool for locating genes controlling important traits of interest such as avirulence, toxin production, host specificity and mating type. Molecular linkage maps can be used to develop molecular markers linked to such traits and ultimately permit the positional cloning of the genes that control them. Genetic linkage maps of other fungal genomes have been constructed using restriction fragment length polymorphisms (RFLPs), random amplified polymorphic DNA (RAPDs), amplified fragment length polymorphisms (AFLPs), microsatellite or simple sequence repeat (SSR) markers, and diversity array technology (DArT) in ascomycetes [8-13], basidiomycetes [14-17], and in oomycetes [18,19]. A preliminary genetic map has been developed for *V. inaequalis *[20], composed of 30 RAPD markers that were divided into six linkage groups, covering 270 cM of the *V. inaequalis *genome. The mating-type (*MAT*) locus was mapped in that population and was flanked by two RAPD markers at 28.9 and 18.9 cM.

In this study, we performed a wide cross under *in vitro *conditions between an isolate of *V. inaequalis *from China and one from the UK to obtain a genetically diverse mapping population of ascospore progeny isolates. We scored this mapping population with AFLP and SSR markers and the mating type (*MAT*) locus and generated a more extensive genetic linkage map than the previously published map of *V. inaequalis*. The map we present covers a genetic distance of 1106 cM over eleven linkage groups and is the first map of *V. inaequalis *to contain transferable SSR loci.

## Methods

### Isolates crossing and obtaining single ascospore isolates and DNA extraction

A population of ascospore progeny isolates from a cross between isolates C0154 (from cv. Qinguan in China) and 01/213 (from cv. Worcester in the UK) was raised following published methods [7], and 83 single-ascospore progeny isolates were produced. The progeny was denoted Q × W for ease of reference. Mycelium was produced *in vitro *on cellophane discs following previously published methods [7]. DNA was extracted from the progeny and parental isolates using the DNeasy Plant Mini Kit following the manufacturer's recommendations and diluted to 10 ng/μl for use in PCR.

### Screening isolates for AFLP and SSR markers

A total of 125 ng of template DNA was analysed using the AFLP analysis system II (Invitrogen, Crawley, UK) according to the manufacturer's protocol and screened with 18 *MseI *+ 1/*EcoRI *+ 2 primer combinations (Table 1) with the *Mse*I primers fluorescently labelled with 6-FAM, VIC or NED (Applied Biosystems, Warrington, UK, Table 1). Sizes of undiluted PCR products were determined using an ABI 3100 genetic analyzer running GENESCAN and GENOTYPER software using GS500LIZ ladder as the internal size standard (Applied Biosystems, Warrington, UK). All segregating AFLP amplicons between 50 and 450 bp were scored, except for primer pairs H (50–400 bp) and K (50–500 bp) (Table 1).

**Table 1:** AFLP primer pairs used for map construction

AFLP primer pairs		Polymorphic bands			Markers with distorted segregation from 1:1^a^			Mapped markers		
Code	Composition	Q	W	Total	P 0.05	P < 0.01	P < 0.001	Q	W	Total
F	M^b^+C/E^c^+AG^d^	17	13	30	11	11	9	9	10	19
H	M+C/E+AT	18	24	42	20	19	12	9	10	19
K	M+C/E+TG	19	17	36	15	11	10	13	10	23
L	M+G/E+TG	18	19	37	27	26	22	3	8	11
M	M+G/E+AG	7	11	18	11	8	6	4	10	14
N	M+G/E+AT	25*** ^e^	5	30	17	13	10	6	4	10
O	M+A/E+AG	15	8	23	16	15	14	4	6	10
P	M+A/E+AT	14*	4	18	13	13	8	11	4	15
R	M+T/E+AG	17	17	34	15	13	11	8	10	18
S	M+T/E+AT	21	21	42	24	18	15	14	13	27
T	M+C/E+AA	8	8	16	12	12	9	1	4	5
V	M+C/E+AC	16	14	30	18	9	6	10	13	23
W	M+A/E+TG	8	5	13	9	8	7	4	0	4
X	M+T/E+TG	14	19	33	18	16	12	8	10	18
Y	M+A/E+AA	8	12	20	15	13	11	5	5	10
Z	M+T/E+AA	23	12	35	20	19	16	9	7	16
AA	M+A/E+AC	16	14	30	16	13	13	12	12	24
BB	M+T/E+AC	16	15	31	18	14	9	9	8	17
	Total	280	238	518	295	251	200	139	144	283

Table 1 lists the AFLP primer pairs used for screening apple scab isolates, and segregation and mapping data for AFLP markers generated for the construction of the genetic linkage map of *Venturia inaequalis*

^a^: Number of markers with a segregation ratio deviating from the 1:1 ratio based on the exact probability from an one sample binomial test

^b^: M stands for MseI sequence – GATGAGTCCTGAGTAA (5' to 3')

^c^: E stands for EcoR1 sequence – AGACTGCGTACCAATTC (5' to 3')

^d^: FAM was attached to E + AA, E + AC and E + AT; VIC to E + AG and NED to E + TG

^e^: *, *** indicates that the C0154 isolate contributed significantly more alleles to the progeny than the 01/213 isolate at the level of P = 0.05 and 0.001, respectively

Twenty-eight published SSR primer pairs [21,22] were screened for polymorphism between the two parental isolates following the published protocols [23]. A further six SSR primer pairs newly developed at East Malling Research were also screened; only three of the six are mapped: EMVi001b (F-AGACAGACGCGAGGACAGAG, R-CCTGTTGTCTCCTCCTCCAC); EMVi029 (F-ACGAGTCCCAGGTCTCACAG, R-TGTTGACGGTCACGGTGTAT); EMVi032c (F-CGGCACAATAGCCATCAGT, R-GAGAGAGACGGGACGAGATG). PCR was performed using a touchdown protocol [23] from 55–50°C in a final volume of 12.5 μl. Primers were labelled on the forward primer with either 6-FAM or HEX fluorescent dyes (VHBio, Newcastle, UK), and analysed on ABI 3100 genetic analyzer running GENESCAN and GENOTYPER software with GS500LIZ ladder as the internal size standard (Applied Biosystems, Warrington, UK).

### Determination of mating types

Each isolate (including the two parent isolates) was manually crossed with four additional isolates, two from each of the two mating types – inferred from a previous study [6]. The success of each of these 340 crosses was evaluated after 6 months to determine the mating type of each isolate.

### Linkage map construction

Segregating molecular markers and data for the *MAT *locus was coded *a/b *for a haploid population and data were analysed using JOINMAP 4.0 (Kyasma, NL) applying the Kosambi mapping function. Marker placement was determined using a minimum LOD score threshold of 4.0, a recombination fraction threshold of 0.35, ripple value of 1.0, jump threshold of 3.0 and a triplet threshold of 5.0. Only linkage groups containing more than five markers were included in the linkage map presented. The map was constructed using MAPCHART2.2 for Windows [24].

## Results

### AFLP, SSR and mating locus analysis of the mapping population

Eighty-three progeny were obtained from the cross between isolates C0154 × 01/213 (Q × W). Table 1 summarises the AFLP analysis of the mapping population. Eighteen AFLP primer combinations produced a total of 978 scoreable amplicons. A total 518 (53%) of these 978 bands were polymorphic between the two parent isolates. The number of bands polymorphic between the parents ranged from 13 to 42 per primer pair, with an average of 28.8 polymorphic markers per primer pair (Table 1). In total, twelve polymorphic SSR loci, derived from ten primer pairs, were scored in the progeny.

Of the 518 polymorphic bands, 280 were present in parent C0154 (Q) and 238 in parent 01/213 (W). In total, 295 molecular markers deviated from the expected 1:1 segregation ratio in the progeny at the 5% level of significance (Table 1). From the 340 pairwise crosses, mating types could only be unambiguously determined for 53 of the 83 progeny isolates. Assigning the mating type '+' and '-' for the parents C0154 and 01/213, respectively, there were 23 and 30 progeny isolates with the respective mating type of '+' and '-'. The ratio between these two mating types did not deviate significantly from the expected 1:1 ratio (*P *= 0.41).

### Linkage map construction

**Figure 1:**
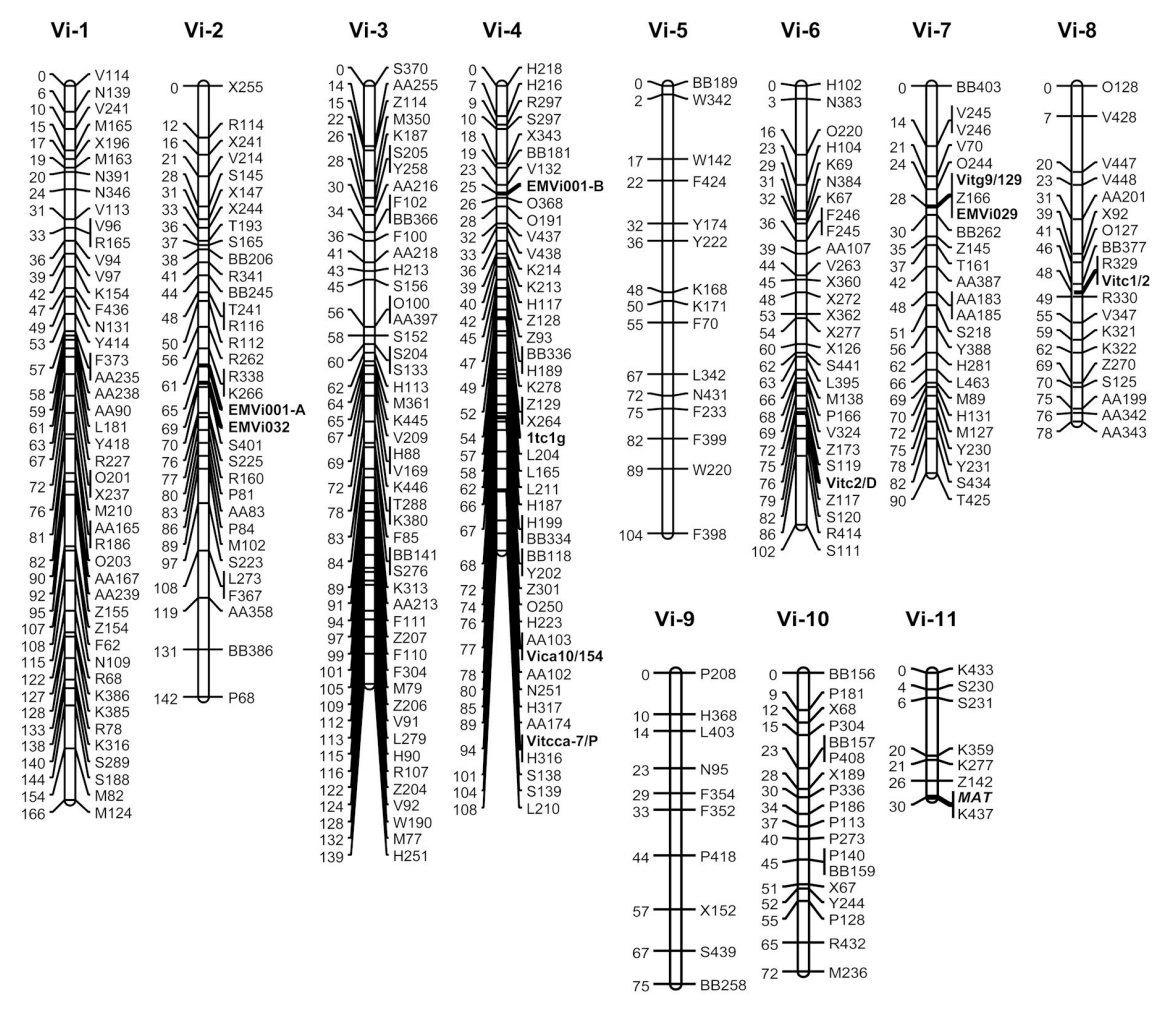
**Linkage map for *Venturia inaequalis***. Figure 1 shows a linkage map for a *Venturia inaequalis *progeny consisting of 83 ascospore progeny isolates derived from a cross between a UK and a Chinese isolate. The map is composed of 294 markers (283 AFLPs, ten SSRs from nine primer pairs, and the *MAT *locus in 11 linkage groups), covering a total genetic distance of 1106 cM.

Analysis of the 531 loci (518 AFLP markers, 12 SSR loci from 10 SSR primer pairs and the mating locus *MAT*) resulted in a genetic map of *V. inaequalis*, consisting of 11 linkage groups, covering a total genetic distance of 1106 cM (Figure 1). A total 294 markers (283 AFLPs, ten SSRs from nine primer pairs, and the *MAT *locus) were mapped onto the 11 linkage groups. Of the 283 AFLP markers mapped, 120 exhibited transmission distortion at the 5% level of significance (Table 1). The two parents contributed a similar number of AFLP markers to the map: 139 and 144 from C0154 (Q) and 01/213 (W), respectively (Table 1).

The length of individual linkage groups ranged from 30.4 cM (Vi-11) to 166 cM (Vi-1). The number of molecular markers per linkage group ranged from 7 on Vi-11 to 48 on Vi-3; the average distance between two loci within each group ranged from 2.4 cM (Vi-4) to 7.5 cM (Vi-9). The maximum map length between two markers within a linkage group was 15.8 cM. The number of mapped AFLP markers ranged from 5 to 27 per primer pair (average = 15.7). Ten of the 12 SSR loci mapped to 5 linkage groups (Vi-2, Vi-4, Vi-6, Vi-7 and Vi-8). The *MAT *locus was mapped to the smallest linkage group (Vi-11), flanked by two AFLP markers at 0.2 cM and 3.9 cM (Figure 1).

## Discussion

We have constructed a genetic linkage map for *V. inaequalis *composed of 294 markers, the majority of which are AFLPs. The total map length is 1106 cM, spanning eleven linkage groups, one of which contains the mating type locus, *MAT*. The map presented is over four times longer than the previously published map of *V. inaequalis *which had a total genetic distance of 270 cM [20] and was composed of just 30 RAPD markers. Thus, the linkage map presented here represents a significant improvement over currently published maps for the study of genome structure and organisation, and for mapping genes of economic importance on the *V. inaequalis *genome.

In this study, approximately 42% of AFLP markers mapped exhibited significant deviations from expected 1:1 segregation ratio at the 5% level of significance. Several genomic processes could be responsible for distorted segregation, including the expression of linked lethal genes, non-disjunction during meiosis due to the parents having divergent genomes and chromosome complements [25], or an accidentally biased selection of the ascospores that formed the mapping population [17]. Between 10% and 80% of the markers mapped in populations of other fungi have been shown to exhibit transmission distortion [13,15,17].

In previous studies, markers with highly distorted segregation (*P *< 0.01) have been excluded from mapping studies of *Mycosphaerella fijiensis *[12], but included in maps of *Fusarium *[13], *Heterobasidion annosum *[15], and *Bremia lactucae *[19]. Despite the segregation distortion observed in this investigation, the majority of linkage associations between markers were strongly supported and thus we have included loci displaying distorted segregation ratios as removing them would have almost halved the number of markers available for linkage analysis, and thus reduced the genome coverage of the map.

The two parent isolates used in the present study were from two continents and populations of *V. inaequalis *from these two continents differed significantly in AFLP markers and virulence characteristics [7]. They were chosen as parents of the mapping progeny presented here because of their genetic differentiation which increased the level of polymorphism observed at genetic loci in the progeny and will permit the mapping of a number of distinct virulence factors in the future. However, this genetic differentiation probably contributed to the high degree of segregation distortion observed in markers used for map construction, as greater genetic divergence between the parents of mapping progenies has been shown to be correlated with higher the levels of transmission ratio distortion [26,27]. Overall, segregation distortion was biased towards the parent C0154 from China, possibly suggesting a general fitness benefit for progeny that inherited C0154 alleles. Similar observations were also obtained in progeny from an inter-specific cross between *F. circinatum *and *F. subglutinans *[13], however, the reasons for this unidirectional distortion in the population presented here are not clear at present.

Whilst 518 markers were scored in the progeny produced, just 283 mapped to groups containing more than five molecular markers. Many of the 235 'unmapped' markers were contained in groups of five markers or less and it is probable that, with the addition of further markers through continued mapping efforts, these smaller groups will become linked to the eleven groups presented here, extending and enhancing this *Venturia *linkage map further.

The *MAT *locus was mapped to a small linkage group and was tightly linked to two AFLP markers. The availability of markers linked to the *MAT *locus will enable us to study the relative importance of conidia and ascospores as primary inoculum by comparing the ratio of two mating types in the autumn and early spring. Understanding the relative importance of conidia and ascospores as primary sources of inoculum may enable appropriate sanitation measures to be taken. The two parent isolates chosen in this study were known to differ in their virulence characteristics against several commercial apple cultivars [7]. Several virulence factors may be needed to overcome the 'partial resistance' in susceptible apple cultivars [6,28]. Further research is now underway to investigate and map the virulence factors that each of the parental isolate possesses.

## Conclusion

A genetic linkage map is important for investigating genetics of important traits of plant fungal pathogens such as virulence factors, aggressiveness and toxin production and mating type. The map presented here does not span the entire *V. inaequalis *genome as it is composed of eleven linkage groups, more than would be expected for a fungus with a base chromosome number of *x *= 7. However, this map provides a foundation for further genome characterisation using transferable markers such as SSRs and gene-specific markers, and for the development of sequence-characterised markers linked to virulence characteristics and the *MAT *locus in *V. inaequalis*.

## Competing interests

The authors declare that they have no competing interests.

## Authors' contributions

XX project leader, provided overall planning for the research, performed data analysis and co-wrote the manuscript. TR maintained fungal isolates and carried out AFLP analysis. DB project consultant, mainly in fungal molecular biology. NH developed six new SSRs and participated in screening isolates for SSRs. LG Assisted in developing new SSR markers. DJS carried out PCR reactions, performed linkage analysis and co-wrote the manuscript. All authors read and approved the final manuscript.
